# Development of New Analytical Microwave-Assisted Extraction Methods for Bioactive Compounds from Myrtle (*Myrtus communis* L.)

**DOI:** 10.3390/molecules23112992

**Published:** 2018-11-16

**Authors:** Ana V. González de Peredo, Mercedes Vázquez-Espinosa, Estrella Espada-Bellido, Ana Jiménez-Cantizano, Marta Ferreiro-González, Antonio Amores-Arrocha, Miguel Palma, Carmelo G. Barroso, Gerardo F. Barbero

**Affiliations:** 1 Department of Analytical Chemistry, Faculty of Sciences, University of Cadiz, Agrifood Campus of International Excellence (ceiA3), IVAGRO, Puerto Real, 11510 Cadiz, Spain; ana.velascogope@uca.es (A.V.G.d.P.); mercedes.vazquez@uca.es (M.V.-E.); estrella.espada@uca.es (E.E.-B.); marta.ferreiro@uca.es (M.F.-G.); miguel.palma@uca.es (M.P.); carmelo.garcia@uca.es (C.G.B.); 2 Department of Chemical Engineering and Food Technology, Faculty of Sciences, University of Cadiz, Agrifood Campus of International Excellence (ceiA3), IVAGRO, Puerto Real, 11510 Cadiz, Spain; ana.jimenezcantizano@uca.es (A.J.-C.); antonio.amores@uca.es (A.A.-A.)

**Keywords:** anthocyanins, bioactive compounds, Box–Behnken design, microwave-assisted extraction, myrtle, *Myrtus communis*, phenolic compounds

## Abstract

The phenolic compounds and anthocyanins present in myrtle berries are responsible for its beneficial health properties. In the present study, a new, microwave-assisted extraction for the analysis of both phenolic compounds and anthocyanins from myrtle pulp has been developed. Different extraction variables, including methanol composition, pH, temperature, and sample–solvent ratio were optimized by applying a Box–Behnken design and response surface methodology. Methanol composition and pH were the most influential variables for the total phenolic compounds (58.20% of the solvent in water at pH 2), and methanol composition and temperature for anthocyanins (50.4% of solvent at 50 °C). The methods developed showed high repeatability and intermediate precision (RSD < 5%). Both methods were applied to myrtle berries collected in two different areas of the province of Cadiz (Spain). Hierarchical clustering analysis results show that the concentration of bioactive compounds in myrtle is related to their geographical origin.

## 1. Introduction

*Myrtus communis* L., the common myrtle, is an evergreen shrub that grows spontaneously in the Mediterranean area and in the Middle East. Myrtle berries have a maximum ripening period from October to February. These berries have multiple shapes and colors [1], but are mainly dark blue in color. The ancient Mediterranean populations already used myrtle mainly for ornamental and aromatic purposes [2]. Recent developments in the fields of health and food have markedly increased their interest in natural compounds with antioxidant potential [3]. The extraction of natural antioxidants in fruits is very useful when substituting synthetic antioxidants, which are being restricted because of their potential health risks and side-effects, and their safety has been questioned for a long time [4]. Therefore, nowadays, myrtle has gained greater recognition in the food and medicinal industries due to its potential beneficial effects [5]. Myrtle presents anti-diabetic, anti-inflammatory, anticancer, antioxidant, antihyperglycaemic, antimycotic, and antiseptic properties [6,7]. For example, myrtle oil is recommended for the treatment of respiratory diseases [8], and it is normally taken as an infusion. In addition, essential oils are used in the perfume and cosmetics industries [9]. Despite the aforementioned uses, myrtle is still mainly known for the production of an aromatic liqueur called “Mirto”, which is obtained by alcoholic maceration of its leaves and fruit. This liquor is very popular and traditional in Sardinia, where it is usually served very cold after meals due to its digestive powers [10].

The phenolic compounds and anthocyanins present in myrtle berries are the main contributors to these beneficial health properties. The quantities of phenolic compounds and anthocyanins present in myrtle berries are extraordinarily high [11,12]. The major phenolic compounds identified in myrtle are quercetin 3-*O*-galactoside, quercetin 3-*O*-rhamnoside, myricetin 3-*O*-rhamnoside, quercetin 3-*O*-glucoside, ellagic acid, and myricetin [13,14]. The major anthocyanins identified are delphinidin 3,5-*O*-diglucoside, delphinidin 3-*O*-glucoside, cyanidin 3-*O*-galactoside, cyanidin 3-*O*-glucoside, cyanidin 3-*O*-arabinoside, petunidin 3-*O*-glucoside, delphinidin 3-*O*-arabinoside, peonidin 3-*O*-glucoside, malvidin 3-*O*-glucoside, petunidin 3-*O*-arabinoside, and malvidin 3-*O*-arabinoside [15,16].

For the extraction of bioactive compounds, specifically for the extraction of phenolic compounds and anthocyanins in vegetable matrices, solid–liquid extraction is usually carried out. Microwave-assisted extraction (MAE) is one of the more advanced extraction methods. MAE is widely used as it is a promising green-extraction method that has the advantage of reducing both extraction time and solvent consumption [17,18]. The microwave technique is based on the application of electromagnetic radiation, with a frequency from 0.3 to 300 GHz. This radiation, which leads to rapid and localized heating of the solvent and sample, is based on a direct effect on the molecules through ionic conduction and dipole rotation [19]. The localized heating leads to a pressure build-up within the cells of the sample, resulting in a rapid transfer of the compound of interest from the cells to the extraction solvent [20]. This extraction method has been widely employed for the extraction of phenolic compounds and anthocyanins from a wide variety of vegetable matrices, such as grapes [21], tomatoes [22], and blackberries [23]. In a recent study, MAE has been applied to extract polyphenols, tannins, and flavonoids from myrtle leaves [24]. Based on the previous results obtained from this study and the high content of bioactive compounds in myrtle leaves, the development of new methods for myrtle analyses to improve the quality of final products, such as liqueurs, is required. Besides, this extraction technique has not yet been completely developed and optimized for the extraction of phenolic compounds and anthocyanins from myrtle berries. As in myrtle leaves, its berries are expected to contain an important amount of the same bioactive compounds besides the anthocyanins due to its dark blue color. This approach is of great interest due to the importance of developing techniques for analysis of the raw material to improve the liqueur quality, which is the main use of myrtle.

The efficiency of MAE can be affected by several variables, such as the solvent (volume, composition, pH), the temperature, the time of application, and the power level [25]. For this reason, experimental designs are usually applied in order to study the effects of the different variables and their interactions, and to determine the optimal conditions. In the present study, a Box–Behnken design (BBD) with a response surface methodology (RSM) was chosen [26,27]. It was employed because the number of experiments necessary to provide sufficient information for statistically acceptable results in a BBD is lower than other statistical designs, and it also ensures that each experiment is in the region of interest, avoiding extreme conditions [28].

The aim of the present study was to develop and optimize MAE methods for the extraction of bioactive compounds (phenolic compounds and anthocyanins) in myrtle in order to evaluate the quality of myrtle berries and to study the possible effect of the geographical origin in the total amount of bioactive compounds.

## 2. Results and Discussion

### 2.1. Development of the MAE Methods

A Box–Behnken design was carried out for the development and optimization of the microwave-assisted extraction of both total phenolic compounds and total anthocyanins (as the sum of individual components) in the myrtle berries. Analysis of variance (ANOVA) was carried out to evaluate the effects of the factors and the possible interactions between them. The factors studied in this work were: solvent composition (% methanol in water), solvent pH, extraction temperature, and sample–solvent ratio. The results of this analysis are shown in Table 1 and Table 2 for the total phenolic compounds and total anthocyanins, respectively. The coefficients for the different parameters of the quadratic polynomial equation and their significance (*p*-values) are presented. The factors and/or interactions that showed a *p*-value lower than 0.05 were considered to be significant factors that influenced the response at the selected level of significance (95%).

With regard to the total phenolic compounds (Table 1), the *p*-values for solvent composition and pH were less than 0.05, meaning that these factors had significant effects. The quadratic interactions of solvent composition (X_1_^2^) and pH (X_2_^2^) had a significant effect on the extraction of phenolic compounds. The interactions between factors were not significant (*p*-value > 0.05). Among the linear terms, the most significant factor was the solvent composition (*p*-value < 0.01), and this had a negative effect (b_1_ = −4.39257), which means that the phenolic compounds were extracted more efficiently when the solvent had a low methanol content in water in this range. The pH also had a negative effect (b_2_ = −3.62017), which means that the extraction of phenolic compounds is more favorable at a low pH. Among the quadratic effects, the methanol effect was more significant than the pH effect, and methanol had a negative effect, whereas pH had a positive effect.

In the case of anthocyanins (Table 2), only the linear term temperature (X_3_) had an influence on the response, with a *p*-value < 0.01. With regard to quadratic effects, X_1_^2^ (solvent composition) once again had a significant effect on the extraction. The interaction between the factors’ pH and temperature (X_2_X_3_) had a significant effect. The temperature had a negative effect (b_2_ = −2.56032), which indicates that a decrease in its value led to a higher recovery of anthocyanins. This should be due to the degradation of anthocyanins when high temperatures were used [29]. The quadratic effect for methanol and the interaction between pH-temperature also had positive coefficients.

**Figure 1:**
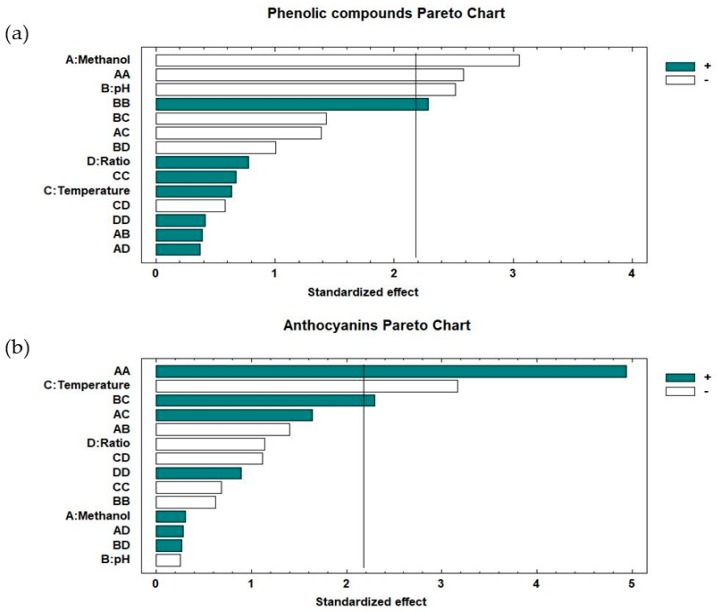
Standardized Pareto charts for: (**a**) total phenolic compounds; (**b**) anthocyanins.

The standardized Pareto chart, which allows for knowledge of the influencing variables and their order of influence from a graphical point of view, is presented in Figure 1. As mentioned earlier, for phenolic compounds (Figure 1a), it can be seen that the significant factors in decreasing order of influence on the response are: methanol percentage, the quadratic interaction of methanol percentage, and the quadratic interaction of pH. For anthocyanins (Figure 1b), the significant factors in the same order are: the quadratic interaction of methanol percentage, the temperature, and the interaction pH temperature.

The complete second-order polynomial model correlates the relationship between independent variables and responses. The correlation was evaluated using the squared correlation coefficients (R^2^). The coefficients obtained for the total phenolic compounds, R^2^ = 77.30%, and the anthocyanins, R^2^ = 82.55%, indicate a statistically significant agreement between the measured and estimated responses. More specifically, the lack-of-fit test showed a *p*-value higher than 0.05 (not significant) for both phenolic compounds and anthocyanins, which means that the models fit well.

The reduced Equations (1) and (2), which show acceptable agreement between the experimental data and the estimated values, are expressed as follows:

Y_TP_ (µg g^−1^) = 42.6799 − 4.3927X_1_ − 3.62017X_2_ − 5.57764X_12_ + 4.93415X_22_ (1)

Y_TA_ (µg g^−1^) = 18.6496 − 2.56032X_3_ + 5.98483X_12_ + 2.3022X_1_X_3_ (2)

**Figure 2:**
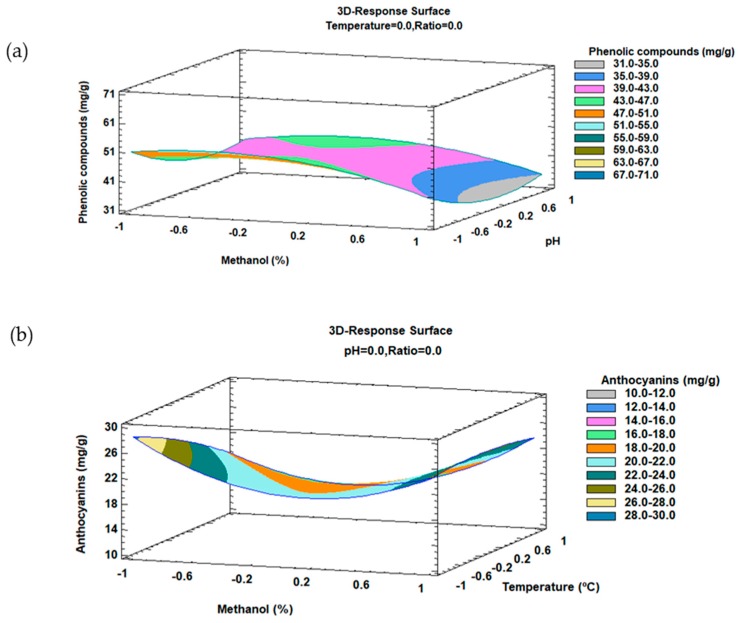
3D surface plots of the Box–Behnken design using polynomial equations: (**a**) solvent composition and pH on the total phenolic compound extraction, and (**b**) temperature and solvent composition on the total anthocyanin extraction.

The trends outlined above were recorded in three-dimensional surface plots obtained by using the polynomial equations. Solvent composition and pH were selected as the most significant factors for phenolic compounds (Figure 2a), and temperature and solvent composition as the most significant factors for anthocyanins (Figure 2b) according to the previous results mentioned above. The plots illustrate the combined effects of the most significant variables on: (**a**) total anthocyanins; and (**b**) total phenolic compound recovery, respectively.

### 2.2. Optimal Conditions

From the Box–Behnken design, it is possible to extract information about the optimum values which show the maximum response for each factor. The optimum MAE conditions to extract the maximum amount of phenolic compounds are as follows: a solvent with 58.20% MeOH in water at pH 2, an extraction temperature of 100 °C, and a 0.5 g/20 mL sample–solvent ratio. The optimum MAE conditions to extract the maximum amount of anthocyanins are as follows: a solvent with 50.4% MeOH in water at pH 3.33, an extraction temperature of 50 °C, and a 0.5 g/20 mL sample–solvent ratio. These results show that the optimal extraction of both phenolic compounds and anthocyanins occurs with values of methanol and pH closer to the lower end of the studied range (50% MeOH in water). With regard to temperature, numerous authors are in agreement that an increase in temperature favors extraction, but also that beyond a certain value, the compounds of interest can be denatured [30]. With respect to anthocyanins, high temperatures may reduce its recovery, since these compounds are thermally sensible and thus can be easily degraded [31]. With respect to phenolic compounds, although anthocyanins are also phenolic compounds, they are present at determinate levels in the overall mixture, so that the other compounds could be different phenolic compounds and less thermally sensible [32]. This possibility would explain why the optimal extraction temperature was high for the total phenolic compounds (100 °C), whereas for the anthocyanins, it was at the lower end of the range studied (50 °C). The phenolic compounds, which were less thermally sensible, increased the solubility in the solvent and the diffusion and mass transfer of the extracted molecules with high temperatures, favoring the extraction [33].

The results obtained at the optimum conditions using MAE were compared with those achieved by other extraction methods already developed from the same raw material (myrtle) [14,34,35,36]. Most of these studies employ traditional extraction techniques that imply long extraction times (in many cases, up to 24 h) without obtaining large recoveries. In comparison with traditional methods, such as maceration, MAE offers better extraction yields of the compounds of interest in a shorter time frame and with lower expense, regarding both solvents and costs. The higher extraction yield of the total phenolic compounds and total anthocyanins could be due to water dipole rotation and ionic conduction effects, which is the main mechanism of microwave heating.

### 2.3. Kinetics of the Extraction Process

**Figure 3:**
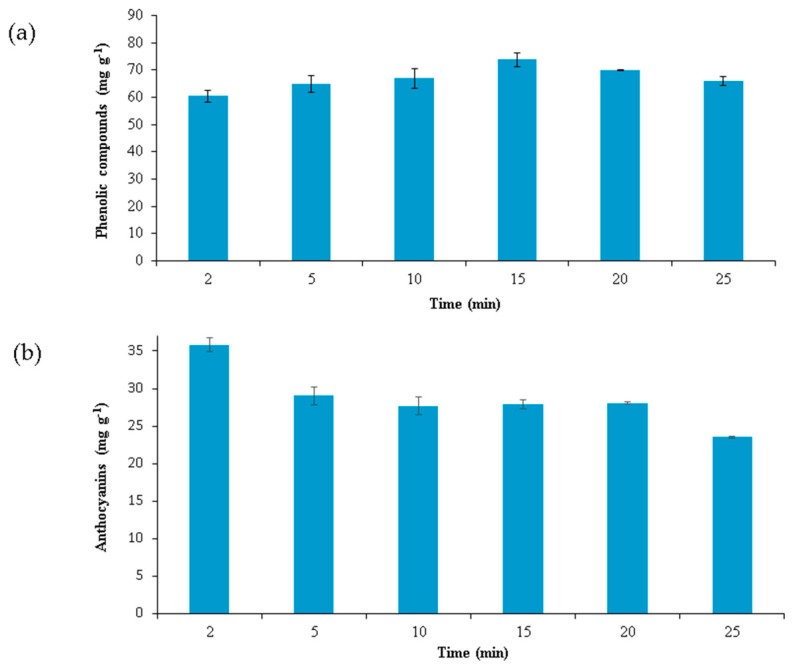
(**a**) Extraction kinetics of total phenolic compounds; (**b**) extraction kinetics of total anthocyanins.

Once the optimal values had been obtained, they were used to study the kinetics of the extraction process. Several extractions were carried out at different times, with fixed values for the factors already studied (percentage of methanol, temperature, sample–solvent ratio, and pH). The experiments were performed in triplicate, and the times employed were: 2, 5, 10, 15, 20, and 25 min. The results for the recovery of total anthocyanins and total phenolic compounds are represented in Figure 3.

It can be seen that for phenolic compounds (Figure 3a), the maximum recovery was achieved at 15 min, being lower when longer times were used. For anthocyanins (Figure 3b), it can be seen that the extracts that were subjected to microwave irradiation for 2 min gave better results—that is, the maximum quantity of anthocyanins was obtained. In addition, a longer extraction time of 5 min gave rise to worse results. This may be due to the degradation of anthocyanins when the extracts are subjected to prolonged microwave irradiation at that temperature [31]. Therefore, for anthocyanins, a shorter time of 2 min was selected as the optimum extraction time, and for phenolic compounds, a longer time of 15 min was chosen. In addition to these optimal extraction times, it is necessary to take into account the extra time required for the extracts to be tempered.

### 2.4. Precision of MAE Methods

The precision of the developed methods was evaluated in terms of repeatability and intermediate precision. Both parameters concern the precision of the MAE of myrtle samples under the same extraction conditions, but repeatability implies extractions carried out on the same day, whereas intermediate precision is related to different days. These terms were evaluated by following the methodology employed in several previous studies [32,37,38]. A total of 30 experiments were developed over three consecutive days by performing ten experiments each day. For repeatability, 10 extractions were performed on the first day of the study. For intermediate precision, 10 more extractions were carried out on each of the next two consecutive days. For phenolic compounds, the repeatability (RSD) was 3.98% and the intermediate precision was 4.54%. For anthocyanins, the repeatability (RSD) was 3.41% and the intermediate precision was 4.10%. Both methods were considered to have good repeatability and intermediate precision, since a maximum error of 5% is generally considered in this type of work [39].

### 2.5. Application to Real Sample

#### 2.5.1. Study of Myrtle Berries from Different Locations

Both MAE methods for total phenolic compounds and for total and individual anthocyanins were applied to the entire myrtle ecotypes collected for this study. A total of 14 different ecotypes of myrtle were evaluated. 8 ecotypes were collected from local evergreen shrubs from the Puerto Real region (My-1, My-2, My-3, My-4, My-5, My-6, My-7, and My-8), and 6 ecotypes were collected from the San José del Valle region (My-9, My-10, My-11, My-12, My-13, and My-14). From each ecotype, the pulp was separated from the seed and processed in duplicate, using the optimum conditions for the developed MAE method for phenolic compounds and the optimum conditions for the extraction of anthocyanins. For the quantification of total phenolic compounds, the extracts were analyzed by the Folin–Ciocalteau (FC) spectrophotometric method. For the quantification of total and individual anthocyanins, the extracts were analyzed by UHPLC. The results of the analyses (total phenolic compounds, the eleven individual anthocyanins, and total anthocyanins) from the 14 myrtle ecotypes are shown in Table 3. First, it is noteworthy to highlight the high content of anthocyanins and total phenolic compounds found in this fruit, equaling or surpassing the contents of well-known superfruits [37,40,41]. The results indicate that myrtle ecotypes collected in the region of Puerto Real have a higher concentration of total phenolic compounds and total anthocyanins than the ecotypes collected in the region of San José del Valle. This information suggests, a priori, that the amount of bioactive compounds in myrtle is related to the location of the ecotype.

#### 2.5.2. Multivariate Statistical Analysis

**Figure 4:**
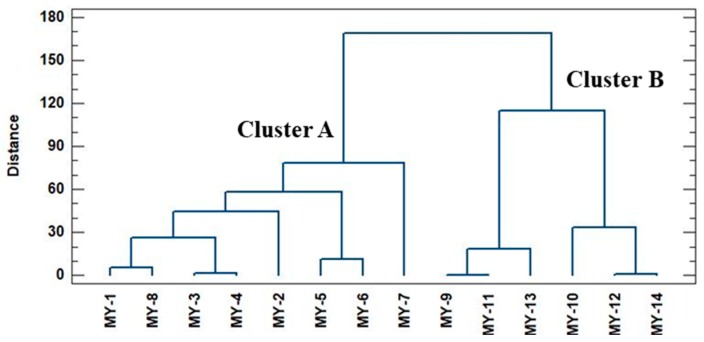
Dendrogram obtained by a hierarchical cluster analysis, based on the chemical parameters studied of the 14 samples by duplicated myrtle pulp extracts.

In order to assess whether the concentration of bioactive compounds in myrtle is related to the location of the ecotype, a non-supervised chemometric technique, namely, hierarchical cluster analysis (HCA), was carried out for the 14 myrtle ecotypes. HCA allows the trends in the myrtle ecotypes to be grouped according to the origin by using the concentrations of the studied bioactive compounds as independent variables for the formation of groups. Therefore, the variables employed in the differentiation were: the amount of total phenolic compounds (mg g^−1^), the amount of each individual anthocyanins (mg g^−1^), and the amount of total anthocyanins (mg g^−1^). Ward´s method was used for the preparation of the clusters, and square Euclidean distance was employed to measure distances between clusters. The results of the HCA are graphically represented in the dendrogram in Figure 4, in which all of the ecotypes of myrtle are listed, along with the distance at which any of the two clusters are joined.

Based on these results, it can be observed that there are two main groups; Cluster A, which includes all of the myrtle ecotypes collected in Puerto Real, and Cluster B, which only includes the myrtle ecotypes collected in San José del Valle. Therefore, it can be concluded that the chemical information obtained—that is, the amounts of total phenolic compounds and anthocyanins, is related to the location of the ecotypes due to the tendency to be grouped according to their geographical origin. Specifically, samples from San José del Valle have a lower number of total phenolic compounds and total anthocyanins than samples collected in Puerto Real. This finding is consistent with bibliographic information, which highlights that myrtle is a shrub that prefers fertile and humid soils, and therefore, warm zones closer to sea level [42,43]. The wet climate of Puerto Real, due to its proximity to the sea, makes the myrtle shrubs of this region mature and grow more favorably than those located in inland areas, such as San José del Valle, where the climate is more variable and dry and the soil is less fertile.

## 3. Material and Methods

### 3.1. Myrtle Sample

Myrtle ecotypes were collected by the authors in December 2016 from local myrtle shrubs in their optimum ripeness stage from two areas (Puerto Real and San José del Valle) of the province of Cadiz, Andalusia, Spain. Specifically, 14 different myrtle ecotypes were collected: 8 ecotypes from the Puerto Real region, and 6 ecotypes from the San Jose del Valle region. Both regions are in the province of Cadiz but have different climatic characteristics, though they are fundamentally based in proximity to the sea. The area of Puerto Real is located on the coast, whereas the region of San José del Valle is located 50 km inland. Proximity to the coast results in very humid areas, with soils that have readily available water, and these provide more fertile conditions for the growth and maturation of many species. By contrast, San José del Valle, as an inland zone, experiences greater temperature changes and less water availability for plants, particularly in the summer. All this leads to the generation of myrtle ecotypes with different characteristics. In addition, a morphological characterization was made of both leaves and berries, applying the guidelines described by M., Mulas & M.R. Cani [43], to confirm that the samples collected came from different ecotypes.

The seeds of the myrtle berries were separated from the pulp. The pulp was lyophilized in a Virtis Benchtop K freeze-drier (SP Scientific, New York, NY, USA) and triturated in a spice grinder. The triturated and homogeneous sample was stored in a freezer at −20 °C prior to analysis.

### 3.2. Chemicals and Solvents

Methanol (HPLC grade) was purchased from Fischer Chemical (Loughborough, United Kingdom). Water was obtained from a Milli-Q water purification system from Millipore (Bedford, MA, USA). Hydrochloric acid and sodium hydroxide (both analytical grade) employed for the adjustment of pH were obtained from Panreac (Barcelona, Spain). The reagents necessary for the determination of total phenolic compounds were anhydrous sodium carbonate (Panreac, Barcelona, Spain), and Folin–Ciocalteu reagent (Merck Millipore, Darmstadt, Germany). The phenolic standard (gallic acid) and the anthocyanin standard (cyanidin chloride) were purchased from Sigma-Aldrich Chemical Co. (St. Louis, MO, USA).

### 3.3. Microwave-Assisted Extraction Procedure

The extraction of total phenolic compounds and total anthocyanins (as the sum of the individual components) from myrtle was performed by microwave-assisted extraction. The extracts were obtained using a MARS 6 One TouchTM Technology system (1800 W) (CEM Corporation, Matthews, NC, USA). Approximately 0.5 g of triturated myrtle was weighed into a MARSXpress vessel (CEM Corporation), and the appropriate volume of solvent was added. The vessel was closed securely and placed in the microwave with another eight tubes, which had the same solvent and volume. Each extraction was carried out under controlled MAE conditions. The parameters used were: solvent composition (50, 75, and 100% methanol in water), pH (2, 4.5, and 7), temperature (50, 75, and 100 °C) and sample–solvent ratio (0.5 g/10 mL, 0.5 g/15 mL, and 0.5 g/20 mL). The initial extraction time was 5 min, and this was followed by a set time to temper the sample. Once the samples had been warmed, the extracts were centrifuged (7500 rpm, 5 min) and the supernatant was added to a 25 mL volumetric flask. The precipitates from the extraction were subsequently redissolved in 5 mL of the same extraction solvent. The extracts were centrifuged again (7500 rpm, 5 min) and the supernatant was placed in the volumetric flask (25 mL). The volume was completed with the same solvent. The extracts were stored at −20 °C prior to analysis.

### 3.4. Identification of Anthocyanins

A chromatographic method using ultra-high performance liquid chromatography (UHPLC) coupled to quadrupole-time-of-flight mass spectrometry (Q-ToF-MS) (Xevo G2 QToF, Waters Corp., Milford, MA, USA) was developed for the identification of anthocyanins in MAE extracts. The injection volume was set to 3 μL. The chromatographic separation was performed on a reverse-phase C18 analytical column (1.7 μm, 2.1 mm × 100 mm, Acquity UPLC BEH C18, Waters). A gradient method, using acidified water (2% formic acid, solvent A) and methanol (solvent B) at a flow rate of 0.4 mL min^−1^ was used. The gradient was as follows (time, % solvent B): 0.00 min, 15%; 3.30 min, 20%; 3.86 min, 30%; 5.05 min, 40%; 5.35 min, 55%; 5.64 min, 60%; 5.95 min, 95%; 7.50 min, 95%. The total run time was 12 min, including 4 min for re-equilibration. The analyses were carried out using an electrospray source operating in positive ionization mode under the following conditions: desolvation gas flow = 700 L h^−1^, desolvation temperature = 500 °C, cone gas flow =10 L h^−1^, source temperature = 150 °C, capillary voltage = 700 V, cone voltage = 30 V and collision energy = 20 eV. The full-scan mode was used (*m*/*z* 100–1200). The following eleven anthocyanins were identified in the samples: delphinidin 3,5-*O*-diglucoside (*m*/*z* 627.1561), delphinidin 3-*O*-glucoside (*m*/*z* 465.1033), cyanidin 3-*O*-galactoside (*m*/*z* 449.1084), cyanidin 3-*O*-glucoside (*m*/*z* 449.1084), cyanidin 3-*O*-arabinoside (*m*/*z* 419.0978), petunidin 3-*O*-glucoside (*m*/*z* 479.1189), delphinidin 3-*O*-arabinoside (*m*/*z* 435.0927), peonidin 3-*O*-glucoside (*m*/*z* 463.1240), malvidin 3-*O*-glucoside (*m*/*z* 493.1346), petunidin 3-*O*-arabinoside (*m*/*z* 449.1084), and malvidin 3-*O*-arabinoside (*m*/*z* 463.1240). Prior to chromatographic analysis, the extracts were filtered through a 0.20 μm nylon syringe filter (Membrane Solutions, Dallas, TX, USA). Information regarding the mass spectra, theoretical and measured masses, as well as the structure of the compounds, are included as Supplementary Material (Table S1 and Figure S1).

### 3.5. Determination of Anthocyanins

**Figure 5:**
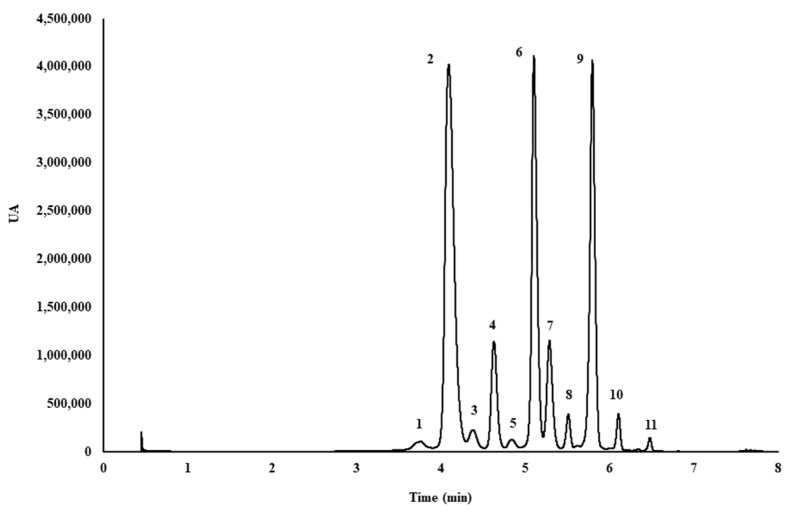
Ultra-high performance liquid chromatography (UHPLC) chromatogram of the eleven anthocyanins identified in the microwave-assisted extraction (MAE) extracts from myrtle berries. Peak assignment: (**1**) delphinidin 3,5-*O*-diglucoside; (**2**) delphinidin 3-*O*-glucoside; (**3**) cyanidin 3-*O*-galactoside; (**4**) cyanidin 3-*O*-glucoside; (**5**) cyanidin 3-*O*-arabinoside; (**6**) petunidin 3-*O*-glucoside; (**7**) delphinidin 3-*O*-arabinoside; (**8**) peonidin 3-*O*-glucoside; (**9**) malvidin 3-*O*-glucoside; (**10**) petunidin 3-*O*-arabinoside; (**11**) malvidin 3-*O*-arabinoside.

Once the anthocyanins had been identified, they were separated and quantified by ultra-high performance liquid chromatography (UHPLC) (Elite LaChrom Ultra System, VWR Hitachi, Tokyo, Japan) available in our research group. The UHPLC chromatogram representing the eleven anthocyanins is shown in Figure 5.

The UHPLC system was equipped with an autosampler (L-2200U), two pumps (L-2160U), a UV-vis detector (L-2420U) set at 520 nm for the analysis, and a column oven (L2300), set at 50 °C for the chromatographic analysis. Anthocyanins were analyzed on a “Fused Core” C18 column (2.6 μm, 2.1 × 100 nm, Phenomenex, Torrance, CA, USA). The separation and quantification were carried out using acidified water (5% formic acid, solvent A) and methanol (solvent B), working at a flow rate of 0.7 mL min^−1^. After testing several methods and flows, amongst other parameters, the gradient employed was as follows (time, % solvent B): 0.00 min, 0%; 1.50 min, 5%; 3.30 min, 15%; 4.80 min, 25%; 5.40 min, 40%. The injection volume was set to 15 µL. This gradient provided optimum results in less than 7 min. Prior to chromatographic analysis, the extracts were filtered through a 0.20 μm nylon syringe filter (Membrane Solutions, Dallas, TX, USA). In order to quantify the eleven anthocyanins present in myrtle extracts, a calibration curve of cyanidin chloride (*y* = 300568.8819*x* − 28462.4337) was used as the anthocyanidin standard. The regression equation and the correlation coefficient (R^2^ = 0.9999) were also calculated using Microsoft Office Excel 2013. The limit of detection (0.196 mg L^−1^) was calculated as three times the standard deviation of the blank signal divided by the slope of the calibration curve. By analogy, the limit of quantification (0.653 mg L^−1^) was calculated as ten times the standard deviation of the blank signal divided by the slope of the calibration curve. The linear range studied was 0.06–35 mg L^−1^. Each one of the eleven anthocyanins was quantified using the calibration curve for cyanidin chloride, and the molecular weight of the anthocyanins analyzed were taken into account. All analyses were carried out in duplicate, and the results were expressed as milligrams of anthocyanins per g of dry weight.

### 3.6. Determination of Total Phenolic Compounds

The Folin–Ciocalteu (FC) spectrophotometric method was used to determine the total phenolic compounds [44,45]. The FC method is based on the fact that phenolic compounds react at basic pH with the Folin–Ciocalteau reagent (a mixture of sodium tungstate and sodium molybdate). The products of the reduction have a blue color, and they have a broad absorption with a maximum of 765 nm. The FC assay was performed by transferring 0.25 mL of MAE extract, 1.25 mL of water, and 1.25 mL of the Folin–Ciocalteu reagent to a volumetric flask (25 mL). Then, 5 mL of a 20% aqueous sodium carbonate solution was also added, and the solution was made up to the mark with water. After 30 min, the absorbance of the solutions was measured at 765 nm. The range of absorbance obtained for the studied samples was 0.4–1.3. Prior to spectrophotometric analysis, the extracts were filtered through a 0.45 µm nylon filter (Membrane Solutions, Dallas, TX, USA). The absorbance was measured on a Heλios-γ Unicam UV-Vis Spectrophotometer (Thermo Scientific, Waltham, MA, USA). In order to quantify the phenolic compounds present in myrtle extracts, a calibration curve was developed under the same conditions, using gallic acid as the reference standard. The results are expressed as milligrams of gallic acid equivalent per g of fresh weight. The regression equation (*y* = 0.0010*x* + 0.0065) and correlation coefficient (R^2^ = 0.9999) were calculated using Microsoft Office Excel 2013. The linear range studied was 100–2600 mg L^−1^. All analyses were carried out in duplicate.

### 3.7. Optimization Procedure and Data Analyses

The spherical response surface Box–Behnken method was employed for experimental design in the optimization procedure. In this approach, the treatment combinations are at the midpoints of the edges of the process space and at the centre, which ensures that all experiments are in the region of interest [46]. To obtain the significant factors and the optimal MAE conditions, a Box–Behnken design with four factors and three levels for each factor was used: a low level (−1), a medium level (0), and a high level (1).

The four factors (independent variables) used in this work were solvent composition (methanol in water) (X_1_), solvent pH (X_2_), extraction temperature (X_3_), and sample–solvent ratio (X_4_), while the response variables (dependent variables) used were the total amount of phenolic compounds (Y_TP_, mg g^–1^) on the one hand, and the total amount of anthocyanins (Y_T_A, mg g^−1^), as the sum of individual ones, on the other. The experimental design consisted of 27 trials performed in duplicate, with three repetitions at the center point to calculate the pure error sum of squares. The whole experimental design matrix and the results obtained are shown in Table 4. My-9 from the San Jose del Valle location was the myrtle sample used for the optimization procedure.

A quadratic model was used for model construction, and this gave a second-order polynomial equation that correlated the relationship between independent variables and responses (Equation (3)):

(3) $$Y=\;\beta_{0}+\overset{k}{\underset{i=1}{\sum }}\beta_{i}X_{i}+\beta_{ii}\;X_{i}^{2}+\underset{i}{\sum }\overset{k}{\underset{i=1}{\sum }}\beta_{ij}X_{i}X_{j}+r$$

where *Y* represents the aforementioned responses (*Y_TP_* and *Y_TA_*) for the extractions of total phenolic compounds and anthocyanins, respectively; *β_0_* is the model constant; *X_i_* are the independent variables; *β_i_* are the linear coefficients; *β_ij_* are the interactive coefficients; *β_ii_* are the quadratic coefficients; and *r* is the pure error sum of squares.

The statistical significances of the model, lack of fit, and regression terms were evaluated from the analysis of variance (ANOVA). The fitting quality of the polynomial model was evaluated by the determination coefficient (R^2^). All experimental data were compiled by Design Expert software 11 (Trial Version, Stat- Ease Inc., Minneapolis, MN, USA). This software was employed for experimental design, data analysis, and model building.

Hierarchical clustering analysis (HCA) was carried out using the Statgraphic Centurion XVII software (Statgraphics Technologies, Inc., The Plains, VA, USA). Pareto Charts were used to identify factors and combinations of factors that are statistically significant at the selected confidence level (95%) for total phenolic compounds and anthocyanins. Regarding the HCA analysis, the Ward method and the Euclidean square distance were used.

## 4. Conclusions

To the best of our knowledge, this is the first study in which MAE has been optimized for the extraction of bioactive compounds, like phenolic compounds and anthocyanins, from myrtle berries. For total phenolic compounds, the solvent methanol-water (58.20% MeOH in water) and pH (pH 2) were the most influential variables. For the recovery of anthocyanins, methanol composition (50.4% MeOH in water) and temperature (50 °C) were found to be the most efficient variables. The optimum extraction times were 2 and 15 min for phenolic compounds and anthocyanins, respectively. Both of the developed methods showed high repeatability and intermediate precision (RSD < 5%). The methods were successfully applied to different myrtle ecotypes collected in two different geographical areas. HCA analysis showed a correlation between the bioactive compounds studied and the location of the ecotype. Due to these previous findings, myrtle berries from more geographical locations could be interesting for further studies.

Based on these results, MAE (under optimum conditions) can be considered as a suitable technique for the extraction of bioactive compounds in myrtle berries. In addition, this technique presents several advantages in comparison to other extraction techniques, since it is fast, economic, and eco-friendlier, as it does not require the use of a high amount of solvent.

## Figures and Tables

**Table 1:** Analysis of variance of the quadratic model adjusted to the extraction of total phenolic compounds. The studied ranges for each parameter were: methanol (50–100%), pH (2–7), temperature (50–100 °C), and sample–solvent ratio (0.5 g/10 mL–0.5 g/20 mL).

Variable	Source	Coefficient	Sum of Squares	Degrees of Freedom	Mean Square	*F*-Value	*p*-Value
	Model		1016.28	14	72.59	2.92	0.0352
Methanol	X_1_	−4.39257	231.54	1	231.54	9.31	0.0101
pH	X_2_	−3.62017	157.27	1	157.27	6.32	0.0272
Temperature	X_3_	0.915742	10.06	1	10.06	0.4047	0.5366
Ratio	X_4_	1.11842	15.01	1	15.01	0.6036	0.4522
Methanol-pH	X_1_X_2_	−5.57764	3.80	1	3.80	0.1528	0.7027
Methanol-Temperature	X_1_X_3_	0.974675	47.75	1	47.75	1.92	0.1911
Methanol-Ratio	X_1_X_4_	−3.45505	3.43	1	3.43	0.1380	0.7167
pH-Temperature	X_2_X_3_	0.926275	51.01	1	51.01	2.05	0.1776
pH-Ratio	X_2_X_4_	4.93415	25.11	1	25.11	1.01	0.3348
Temperature-Ratio	X_3_X_4_	−3.571	8.33	1	8.33	0.3350	0.5734
Methanol-Methanol	X_1_^2^	−2.50565	165.92	1	165.92	6.67	0.0240
pH-pH	X_2_^2^	1.45055	129.84	1	129.84	5.22	0.0413
Temperature-Temperature	X_3_^2^	−1.44313	11.22	1	11.22	0.4513	0.5145
Ratio-Ratio	X_4_^2^	0.889012	4.22	1	4.22	0.1695	0.6878
Residual		42.6799	298.41	12	24.87		
Lack of Fit			266.57	10	26.66	1.67	0.4311
Pure Error			31.83	2	15.92		
Total			1314.68	26			

**Table 2:** Analysis of variance of the quadratic model, adjusted to the extraction of total anthocyanins. The ranges studied for each parameter were: methanol (50–100%), pH (2–7), temperature (50–100 °C), and sample–solvent ratio (0.5 g/10 mL–0.5 g/20 mL).

Variable	Source	Coefficient	Sum of Squares	Degrees of Freedom	Mean Square	*F*-Value	*p*-Value
	Model		445.11	14	31.79	4.05	0.0100
Methanol	X_1_	0.249033	0.7442	1	0.7442	0.0949	0.7633
pH	X_2_	−0.207067	0.5145	1	0.5145	0.0656	0.8022
Temperature	X_3_	−2.56032	78.66	1	78.66	10.03	0.0081
Ratio	X_4_	−0.92215	10.20	1	10.20	1.30	0.2762
Methanol-pH	X_1_X_2_	5.98483	15.51	1	15.51	1.98	0.1850
Methanol-Temperature	X_1_X_3_	−1.9692	21.20	1	21.20	2.70	0.1260
Methanol-Ratio	X_1_X_4_	2.3022	0.6398	1	0.6398	0.0816	0.7800
pH-Temperature	X_2_X_3_	0.39995	41.29	1	41.29	5.27	0.0406
pH-Ratio	X_2_X_4_	−0.758496	0.5627	1	0.5627	0.0718	0.7933
Temperature-Ratio	X_3_X_4_	3.21277	9.76	1	9.76	1.24	0.2864
Methanol-Methanol	X_1_^2^	0.375075	191.03	1	191.03	24.36	0.0003
pH-pH	X_2_^2^	−0.830096	3.07	1	3.07	0.3913	0.5433
Temperature-Temperature	X_3_^2^	−1.56213	3.67	1	3.67	0.4687	0.5066
Ratio-Ratio	X_4_^2^	1.0868	6.30	1	6.30	0.8034	0.3877
Residual		18.6496	94.10	12	7.84		
Lack of Fit			87.43	10	8.74	2.62	0.3076
Pure Error			6.67	2	3.33		
Total			539.21	26			

**Table 3:** Results of total phenolic compounds (mg g^−1^) and total and individual anthocyanins (mg g^−1^) for each myrtle ecotype (*n* = 3). Del-3,5-diGl: delphinidin 3,5-*O*-diglucoside; Del-3-Glu: delphinidin 3-*O*-glucoside; Cy-3-Ga: cyanidin 3-*O*-galactoside; Cy-3-Gl: cyanidin 3-*O*-glucoside; Cy-3-Ar: cyanidin 3-*O*-arabinoside; Pet-3-Gl: petunidin 3-*O*-glucoside; Del-3-Ara: delphinidin 3-*O*-arabinoside; Peo-3-Gl: peonidin 3-*O*-glucoside; Mal-3-Gl: malvidin 3-*O*-glucoside; Pet-3-Ar: petunidin 3-*O*-arabinoside; Mal-3-Ar: malvidin 3-*O*-arabinoside.

Compounds	Myrtle Ecotypes of Puerto Real								Myrtle Ecotypes of San José del Valle					
	MY-1	MY-2	MY-3	MY-4	MY-5	MY-6	MY-7	MY-8	MY-9	MY-10	MY-11	MY-12	MY-13	MY-14
Del-3,5-diGl	0.434 ± 0.012	0.440 ± 0.015	0.367 ± 0.013	0.352 ± 0.035	0.514 ± 0.0132	0.397 ± 0.018	0.210 ± 0.001	0.498± 0.023	0.156 ± 0.004	0.073 ± 0.003	0.1456 ± 0.004	0.181 ± 0.005	0.157 ± 0.004	0.180 ± 0.007
Del-3-Glu	9.405 ± 0.256	13.232 ± 0.369	9.555 ± 0.051	9.704 ± 0.159	15.110 ± 0.160	10.935 ± 0.001	10.164 ± 0.171	9.664 ± 0.497	8.049 ± 0.283	1.798 ± 0.072	7.897 ± 0.301	6.102 ± 0.234	5.432 ± 0.234	6.192 ± 0.236
Cy-3-Ga	0.159 ± 0.0006	0.288 ± 0.014	0.211 ± 0.010	0.309 ± 0.010	0.372 ± 0.010	0.326 ± 0.003	0.184 ± 0.004	0.426 ± 0.023	0.504 ± 0.023	0.047 ± 0.001	0.490 ± 0.023	0.133 ± 0.003	0.341 ± 0.013	0.154 ± 0.005
Cy-3-Gl	1.702 ± 0.063	2.326 ± 0.075	1.013 ± 0.019	1.067 ± 0.022	1.854 ± 0.048	1.142 ± 0.015	2.276 ± 0.016	1.626 ± 0.079	1.011 ± 0.039	0.321 ± 0.013	1.002 ± 0.035	1.191 ± 0.043	0.988 ± 0.038	1.235 ± 0.031
Cy-3-Ar	0.090 ± 0.002	0.098 ± 0.002	0.075 ± 0.002	0.145 ± 0.009	0.136 ± 0.0014	0.611 ± 0.656	2.124 ± 0.015	0.134 ± 0.005	0.943 ± 0.037	0.299 ± 0.012	0.898 ± 0.032	0.084 ± 0.002	0.765 ± 0.029	0.085 ± 0.038
Pet-3-Gl	4.738 ± 0.065	8.680 ± 0.239	6.512 ± 0.027	7.480 ± 0.159	9.958 ± 0.426	4.614 ± 0.196	2.036 ± 0.023	6.058 ± 0.330	0.094 ± 0.010	0.036 ± 0.001	0.091 ± 0.003	2.346 ± 0.087	1.247 ± 0.051	2.988 ± 0.002
Del-3-Ara	1.979 ± 0.073	1.838 ± 0.072	1.527 ± 0.069	1.601 ± 0.032	2.320 ± 0.062	4.974 ± 0.234	4.022 ± 0.052	1.627 ± 0.079	6.256 ± 0.222	1.634 ± 0.072	5.990 ± 0.189	0.900 ± 0.023	3.257 ± 0.138	0.912 ± 0.119
Peo-3-Gl	0.614 ± 0.021	0.856 ± 0.027	0.573 ± 0.023	0.608 ± 0.022	0.705 ± 0.019	0.7206 ± 0.056	0.869 ± 0.052	0.406 ± 0.022	0.627 ± 0.022	0.330 ± 0.011	0.599 ± 0.019	0.488 ± 0.023	0.178 ± 0.07	0.375 ± 0.029
Mal-3-Gl	6.893 ± 0.245	13.124 ± 0.417	14.346 ± 0.024	15.495 ± 0.331	0.750 ± 0.020	0.767 ± 0.060	0.925 ± 0.0004	6.227 ± 0.339	0.667 ± 0.024	0.352 ± 0.013	0.601 ± 0.023	9.832 ± 0.342	5.563 ± 0.190	6.877 ± 0.013
Pet-3-Ar	0.295 ± 0.0003	0.430 ± 0.010	0.411 ± 0.0174	0.504 ± 0.020	0.590 ± 0.013	0.656 ± 0.021	0.3891 ± 0.0004	0.378 ± 0.018	0.765 ± 0.023	0.203 ± 0.001	0.679 ± 0.021	0.184 ± 0.007	0.634 ± 0.022	0.199 ± 0.268
Mal-3-Ar	0.2451 ± 0.002	0.231 ± 0.007	0.356 ± 0.002	0.372 ± 0.002	0.328 ± 0.006	0.5402 ± 0.001	0.289 ± 0.002	0.154 ± 0.010	0.520 ± 0.024	0.263 ± 0.01	0.492 ± 0.012	0.299 ± 0.013	0.5757 ± 0.020	0.213 ± 0.007
Total anthocyanins	26.555 ± 0.395	41.544 ± 1.390	34.947 ± 0.183	37.638 ± 0.730	32.637 ± 0.854	25.682 ± 0.846	23.487 ± 0.234	35.846 ± 0.896	19.595 ± 0.577	5.355 ± 0.242	18.884 ± 0.762	21.740 ± 0.865	19.138 ± 0.645	19.409 ± 0.567
Total phenolic compounds	88.598 ± 2.983	124.684 ± 0.934	82.603 ± 0.343	63.457 ± 2.541	86.251 ± 2.934	81.225 ± 2.199	88.340 ± 3.899	90.682 ± 4.706	55.934 ± 2.743	69.550 ± 3.123	56.790 ± 2.065	60.654 ± 2.127	61.193 ± 2.356	59.898 ± 2.967

**Table 4:** Box–Behnken design matrix of four variables, and measured and predicted responses.

Run	Factors				Responses			
	Solvent	pH	Temp.	Ratio	Y_TP_ (mg g^−1^)		Y_TA_ (mg g^−1^)	
					Measured	Predicted	Measured	Predicted
1	−1	−1	0	0	50.66	51.02	26.04	21.86
2	1	−1	0	0	34.64	40.29	24.74	26.30
3	−1	1	0	0	43.86	41.83	26.73	25.39
4	1	1	0	0	31.74	35.00	17.54	21.95
5	0	0	−1	−1	44.30	41.54	20.05	20.83
6	0	0	1	−1	42.60	46.26	20.50	18.83
7	0	0	−1	1	46.71	46.67	20.21	22.11
8	0	0	1	1	39.23	45.61	14.41	13.86
9	0	0	0	0	38.17	42.68	16.56	18.65
10	−1	0	0	−1	38.51	42.19	25.34	26.79
11	1	0	0	−1	31.50	31.55	26.42	26.49
12	−1	0	0	1	40.14	42.58	25.04	24.15
13	1	0	0	1	36.83	35.64	27.71	25.45
14	0	−1	−1	0	44.07	48.20	22.79	23.04
15	0	1	−1	0	46.49	48.10	18.40	16.20
16	0	−1	1	0	56.28	57.17	10.11	11.50
17	0	1	1	0	44.42	42.79	18.57	17.51
18	0	0	0	0	44.14	42.68	19.45	18.65
19	0	−1	0	−1	53.28	48.50	20.59	20.48
20	0	1	0	−1	46.14	46.27	19.82	19.32
21	0	−1	0	1	62.00	55.75	16.79	17.89
22	0	1	0	1	44.84	43.50	17.52	18.22
23	−1	0	−1	0	39.22	38.57	26.60	28.42
24	1	0	−1	0	39.00	36.70	26.86	24.31
25	−1	0	1	0	51.14	47.32	15.60	18.69
26	1	0	1	0	37.10	31.62	25.03	23.80
27	0	0	0	0	45.74	42.68	19.94	18.65

## Supplementary Materials

The following are available online, Figure S1: MS spectra and structure of the eleven anthocyanins identified in myrtle berries: (**a**) Delphinidin 3,5-*O*-diglucoside; (**b**) Delphinidin 3-*O*-glucoside; (**c**) Cyanidin 3-*O*-galactoside; (**d**) Cyanidin 3-*O*-glucoside; (**e**) Cyanidin 3-*O*-arabinoside; (**f**) Petunidin 3-*O*-glucoside; (**g**) Delphinidin 3-*O*-arabinoside; (**h**) Peonidin 3-*O*-glucoside; (**i**) Malvidin 3-*O*-glucoside; (**j**) Petunidin 3-*O*-arabinoside; (**k**) Malvidin 3-*O*-arabinoside, Table S1: Mass spectra information of the eleven anthocyanins present in myrtle berries.
