# Supplementary material for: Development of New Analytical Microwave-Assisted Extraction Methods for Bioactive Compounds from Myrtle (Myrtus communis L.)
Source: Molecules. 2018 Nov 16;23(11):2992. doi: 10.3390/molecules23112992 (PMC6278529; doi:10.3390/molecules23112992)
Supplement: Supplementary file 1 [file molecules-23-02992-s001.pdf]

**Table S1:** Mass spectra information of the eleven anthocyanins present in myrtle berries.

| Anthocyanins present in myrtle berries | Molecular formula | Theoretical mass (*m*/*z*) | Measured mass (*m*/*z*) |
|---|---|---|---|
| Delphinidin 3,5-*O*-diglucoside | $C_{27}H_{31}O_{17}$ | 627.1561 | 627.1556 |
| Delphinidin 3-*O*-glucoside | $C_{21}H_{21}O_{12}$ | 465.1033 | 465.1047 |
| Cyanidin 3-*O*-galactoside | $C_{21}H_{21}O_{11}$ | 449.1084 | 449.1089 |
| Cyanidin 3-*O*-glucoside | $C_{21}H_{21}O_{11}$ | 449.1084 | 449.1089 |
| Cyanidin 3-*O*-arabinoside | $C_{20}H_{19}O_{10}$ | 419.0978 | 419.0979 |
| Petunidin 3-*O*-glucoside | $C_{22}H_{23}O_{12}$ | 479.1190 | 479.1195 |
| Delphinidin 3-*O*-arabinoside | $C_{20}H_{19}O_{11}$ | 435.0927 | 435.0931 |
| Peonidin 3-*O*-glucoside | $C_{22}H_{23}O_{11}$ | 463.1240 | 463.1249 |
| Malvidin 3-*O*-glucoside | $C_{23}H_{25}O_{12}$ | 493.1346 | 493.1385 |
| Petunidin 3-*O*-arabinoside | $C_{21}H_{21}O_{11}$ | 449.1084 | 449.1101 |
| Malvidin 3-*O*-arabinoside | $C_{22}H_{23}O_{11}$ | 463.1240 | 463.1238 |

(a)

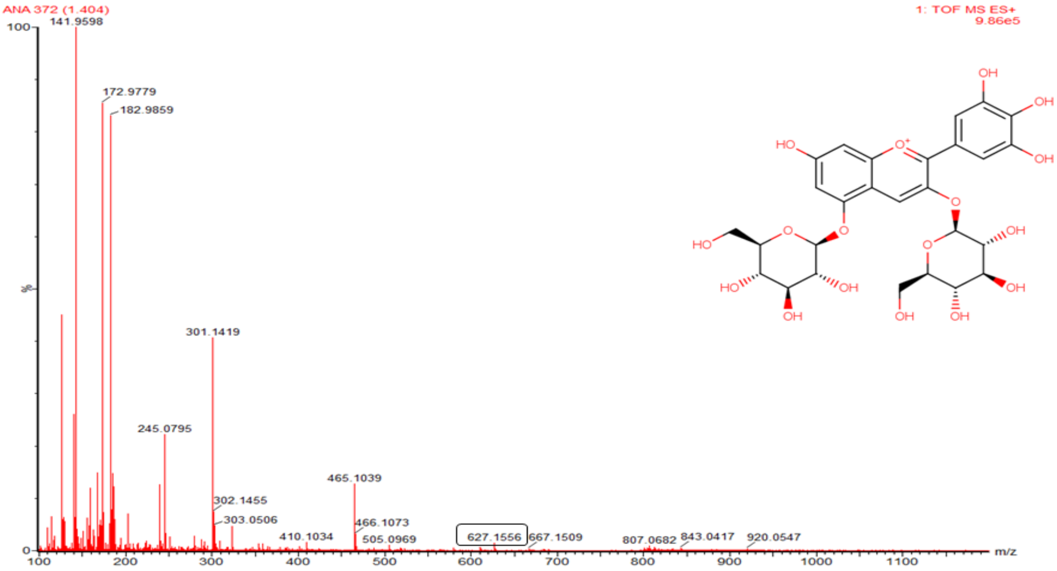

(b)

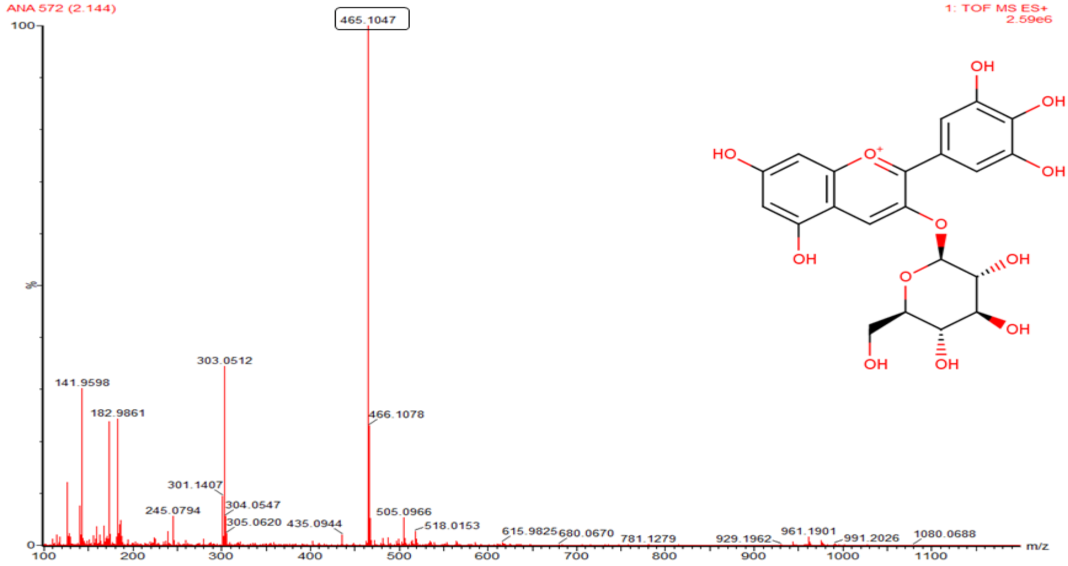

ANA 572 (2.144)
1: TOF MS ES+
2.59e6
465.1047
303.0512
141.9598
182.9861
466.1078
301.1407
304.0547
245.0794
305.0620
435.0944
505.0966
518.0153
615.9825
680.0670
781.1279
929.1962
961.1901
991.2026
1080.0688
m/z
%
OH
OH
OH
HO
OH
O
OH
OH
OH
OH


(c)

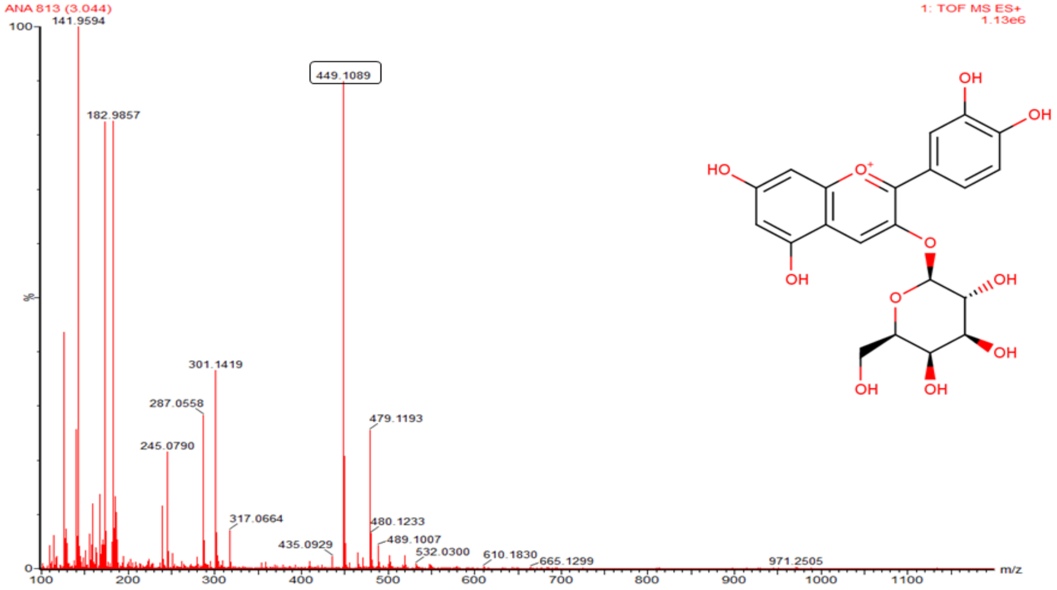

ANA 813 (3.044)
1: TOF MS ES+
1.13e6
141.9594
449.1089
182.9857
301.1419
287.0558
479.1193
245.0790
317.0664
480.1233
435.0929
489.1007
532.0300
610.1830
665.1299
971.2505
m/z
%
OH
OH
HO
OH
O
OH
OH
OH
OH


(d)

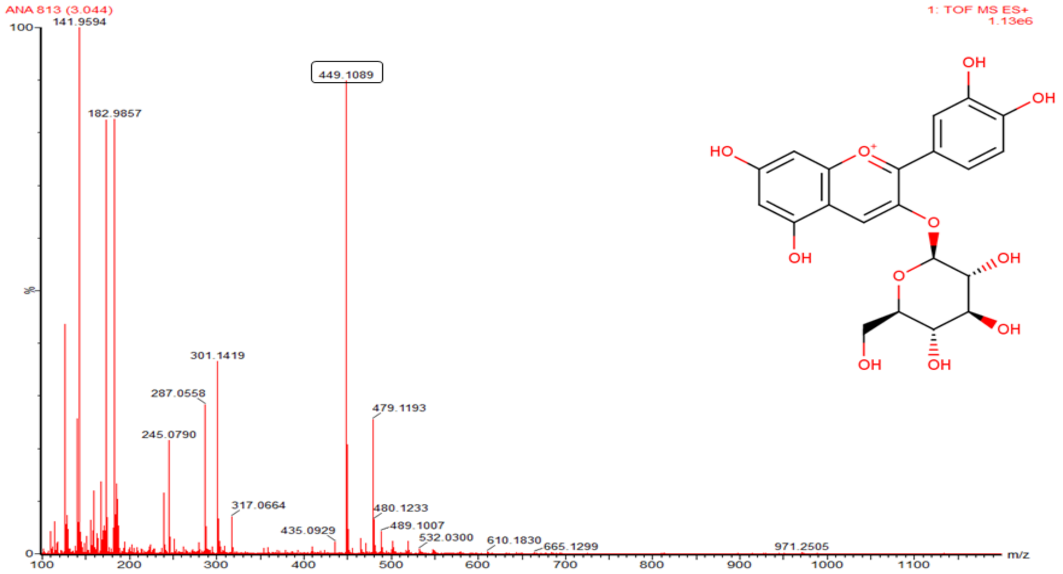

ANA 813 (3.044)
1: TOF MS ES+
1.13e6
141.9594
449.1089
182.9857
301.1419
287.0558
479.1193
245.0790
317.0664
480.1233
435.0929
489.1007
532.0300
610.1830
665.1299
971.2505
m/z
%
OH
OH
HO
OH
O
OH
OH
OH
OH

(e)

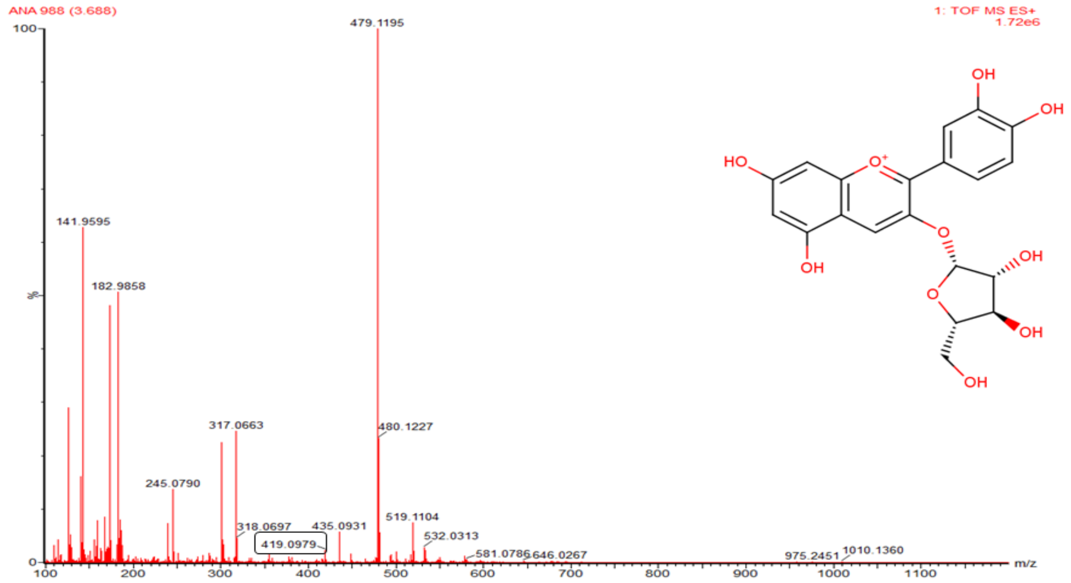

ANA 988 (3.688)
1: TOF MS ES+
1.72e6
479.1195
141.9595
182.9858
317.0663
480.1227
245.0790
519.1104
318.0697
435.0931
419.0979
532.0313
581.0786
646.0267
975.2451
1010.1360
m/z


(f)

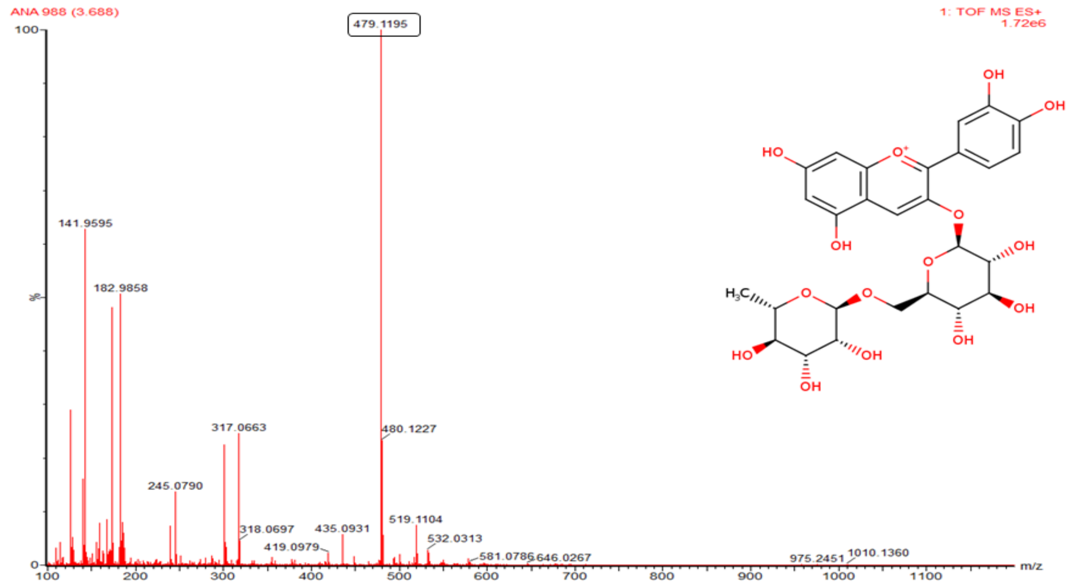

ANA 988 (3.688)
1: TOF MS ES+
1.72e6
479.1195
141.9595
182.9858
317.0663
480.1227
245.0790
519.1104
318.0697
435.0931
419.0979
532.0313
581.0786
646.0267
975.2451
1010.1360
m/z


(g)

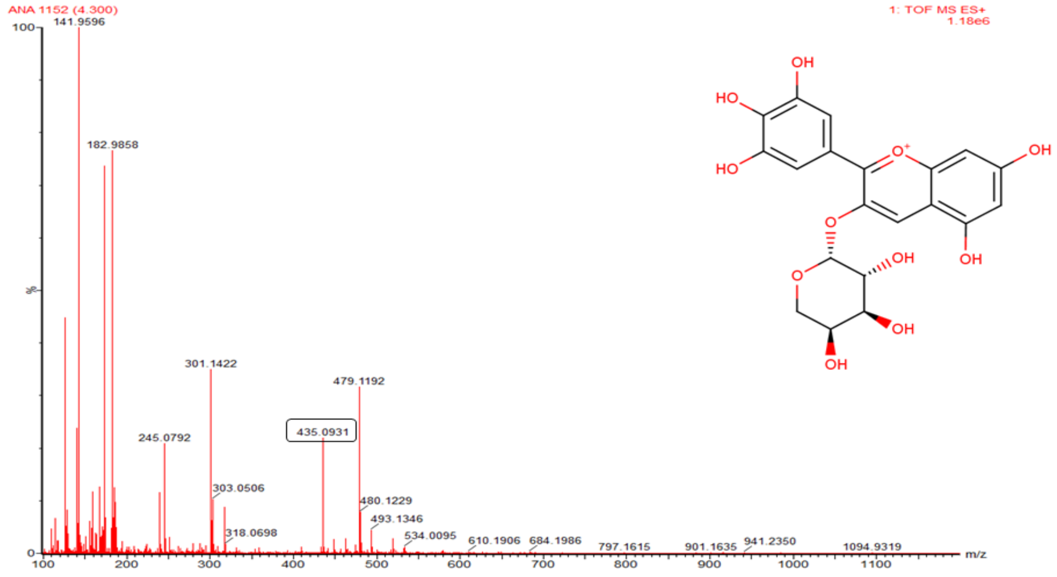

ANA 1152 (4.300)
1: TOF MS ES+
1.18e6
141.9596
182.9858
301.1422
479.1192
245.0792
435.0931
303.0506
480.1229
493.1346
318.0698
534.0095
610.1906
684.1986
797.1615
901.1635
941.2350
1094.9319
m/z

(h)

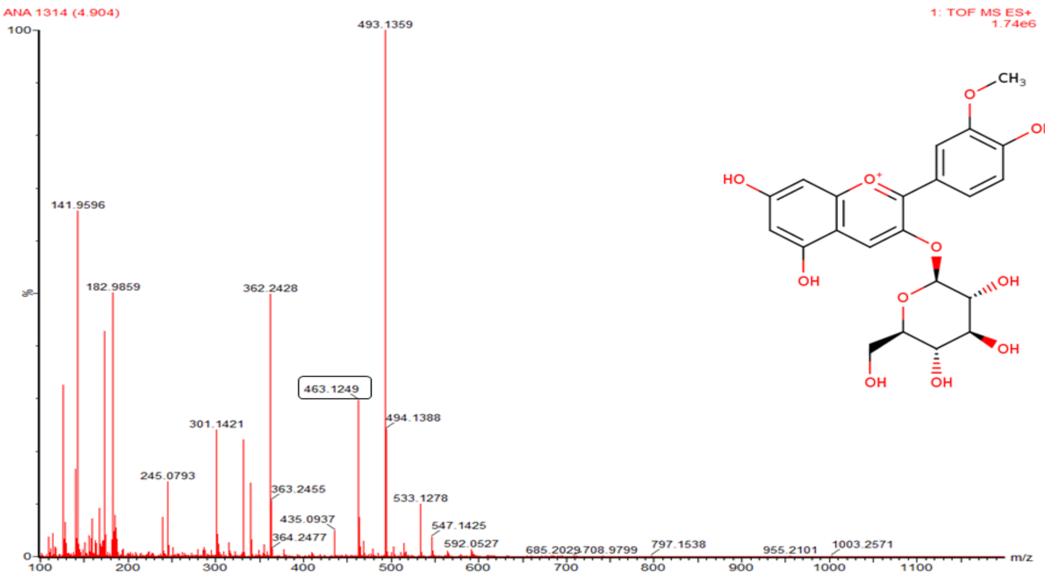

ANA 1314 (4.904)
1: TOF MS ES+
1.74e6
493.1359
141.9596
182.9859
362.2428
463.1249
494.1388
301.1421
245.0793
363.2455
533.1278
435.0937
547.1425
364.2477
592.0527
685.2029
708.9799
797.1538
955.2101
1003.2571
m/z
%
HO
OH
O
CH3


(i)

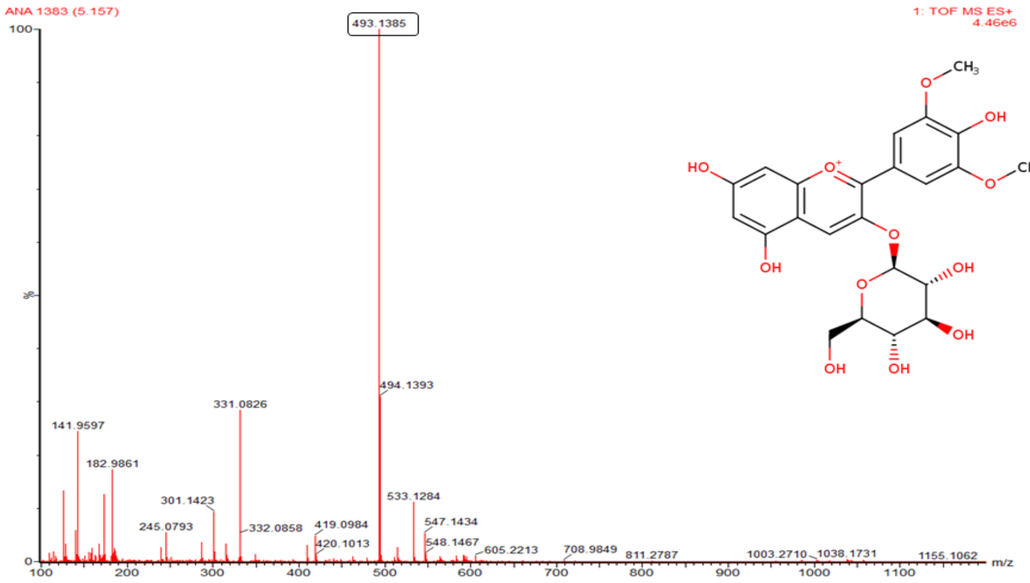

ANA 1383 (5.157)
1: TOF MS ES+
4.46e6
493.1385
494.1393
331.0826
141.9597
182.9861
301.1423
533.1284
245.0793
332.0858
419.0984
547.1434
420.1013
548.1467
605.2213
708.9849
811.2787
1003.2710
1038.1731
1155.1062
m/z
%
HO
OH
O
CH3


(j)

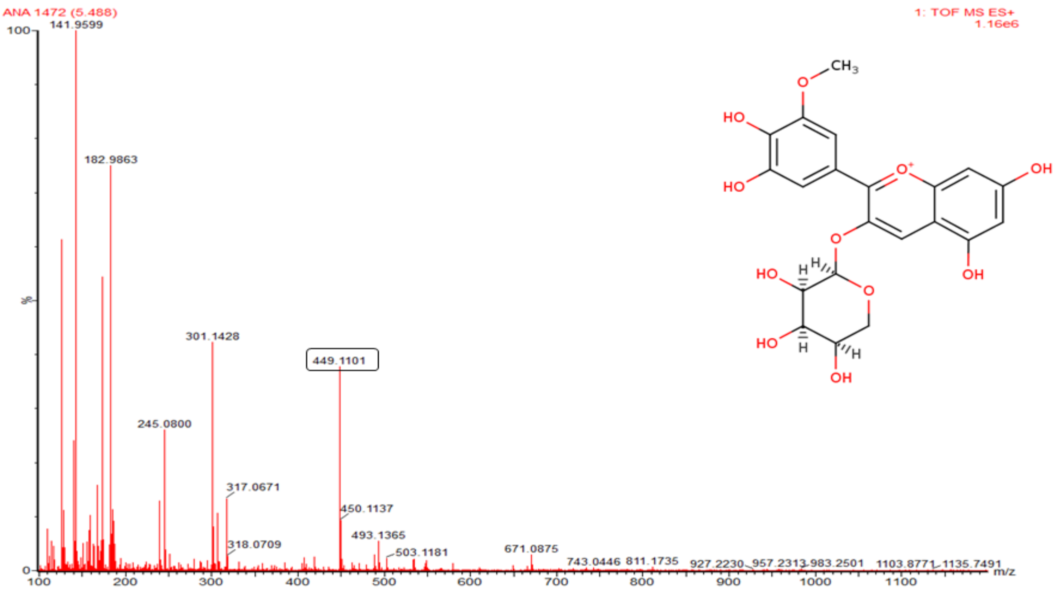

ANA 1472 (5.488)
1: TOF MS ES+
1.16e6
141.9599
182.9863
301.1428
449.1101
245.0800
317.0671
450.1137
318.0709
493.1365
503.1181
671.0875
743.0446
811.1735
927.2230
957.2313
983.2501
1103.8771
1135.7491
m/z
%
HO
OH
O
H
CH3

(k)

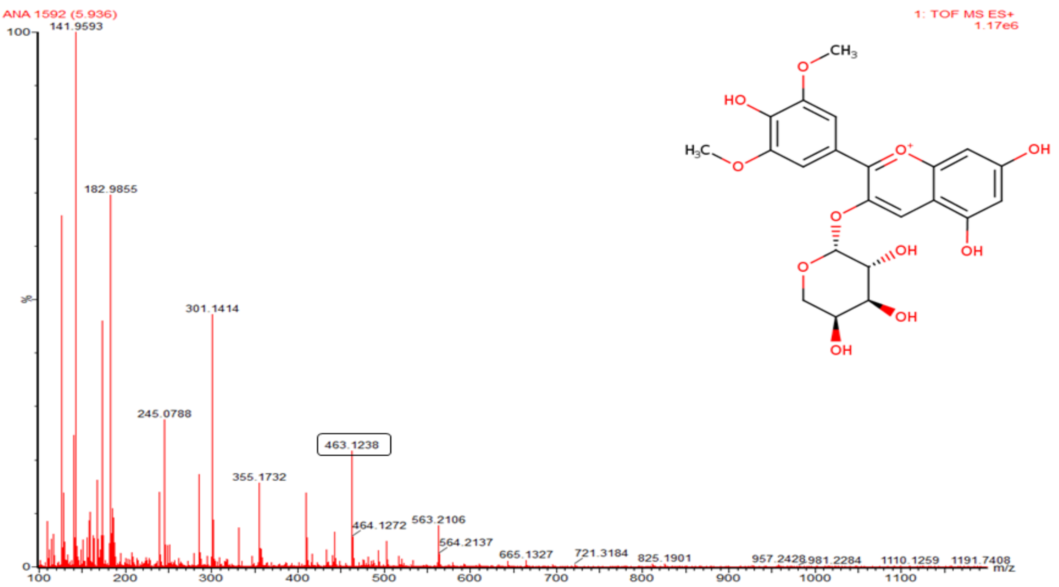


**Figure S1:** MS spectra and structure of the eleven anthocyanins identified in myrtle berries: (a) Delphinidin 3,5-*O*-diglucoside; (b) Delphinidin 3-*O*-glucoside; (c) Cyanidin 3-*O*-galactoside; (d) Cyanidin 3-*O*-glucoside; (e) Cyanidin 3-*O*-arabinoside; (f) Petunidin 3-*O*-glucoside; (g) Delphinidin 3-*O*-arabinoside; (h) Peonidin 3-*O*-glucoside; (i) Malvidin 3-*O*-glucoside; (j) Petunidin 3-*O*-arabinoside; (k) Malvidin 3-*O*-arabinoside.
